# Supplementary material for: Multi‐omics identify xanthine as a pro‐survival metabolite for nematodes with mitochondrial dysfunction
Source: EMBO J. 2019 Feb 22;38(6):e99558. doi: 10.15252/embj.201899558 (PMC6418696; doi:10.15252/embj.201899558)
Supplement: Supplementary file 1 — Appendix [file EMBJ-38-e99558-s001.pdf]

TABLE OF CONTENTS

- **Appendix Figures**
    - Appendix Figure S1
    - Appendix Figure S2
    - Appendix Figure S3
    - Appendix Figure S4

- **Appendix Figure Legends**

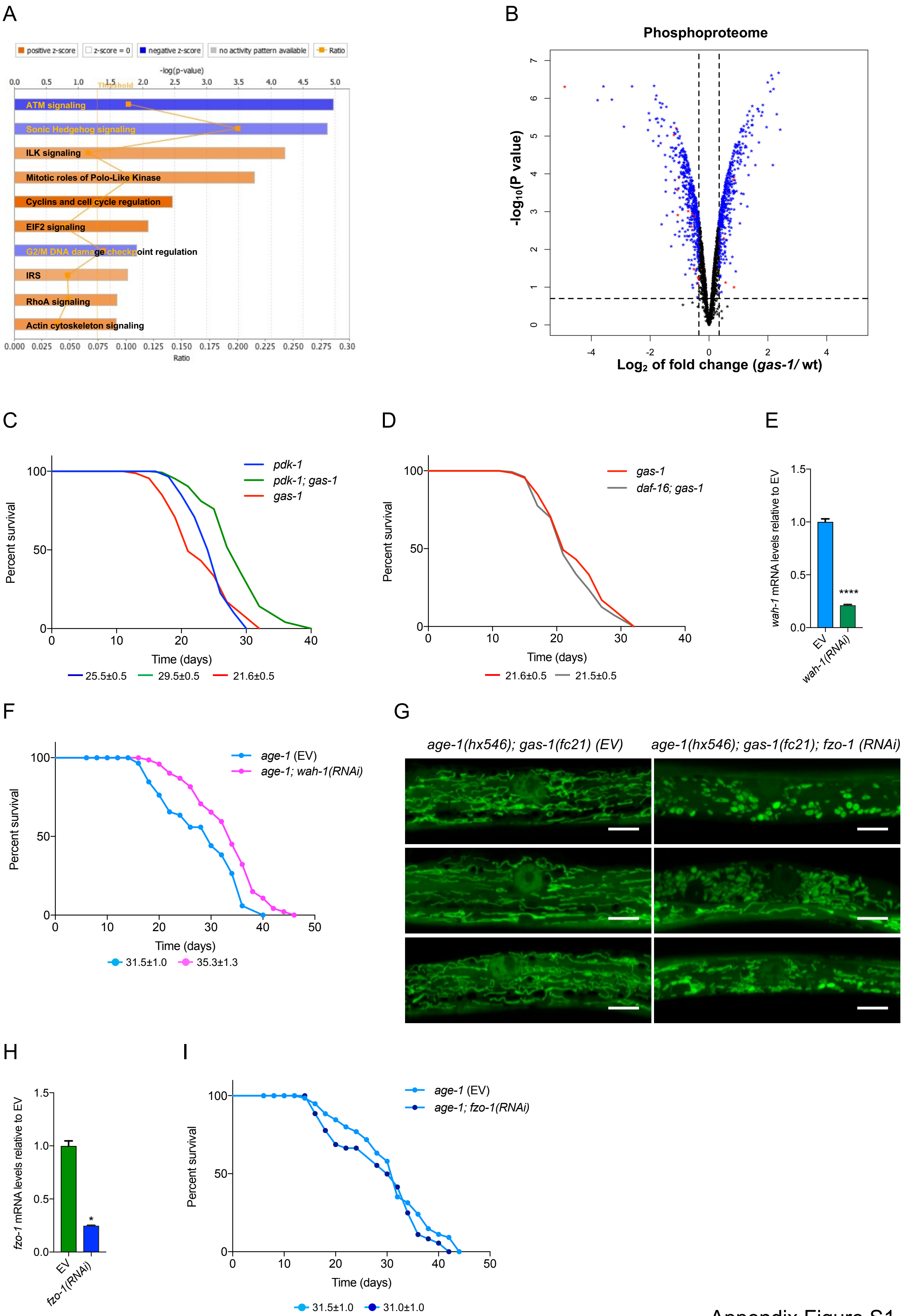

Appendix Figure S1

A

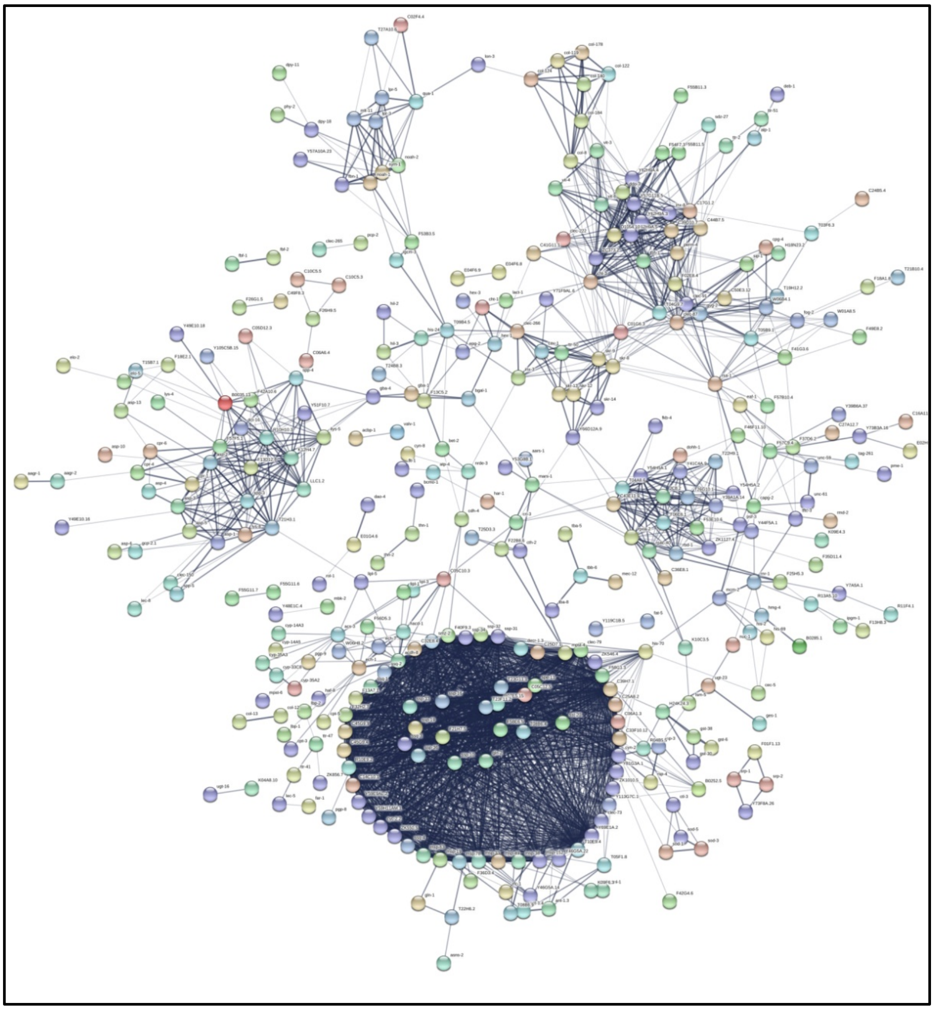

B

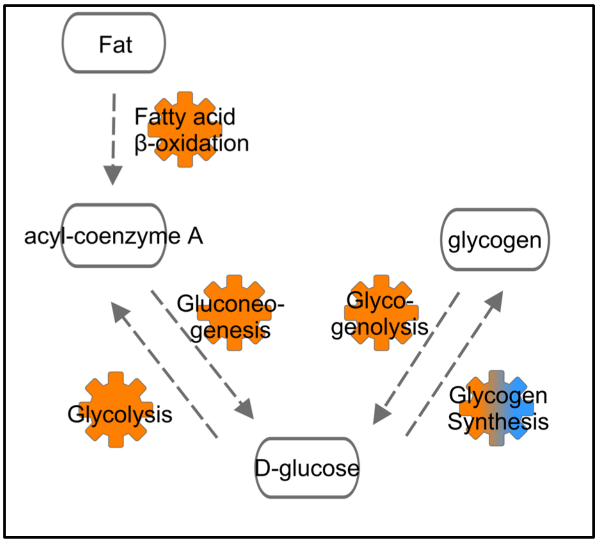

C

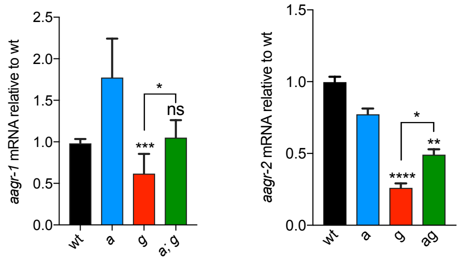

D

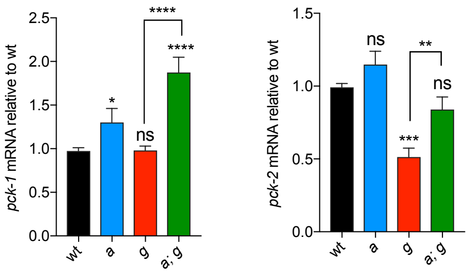

Appendix Figure S2

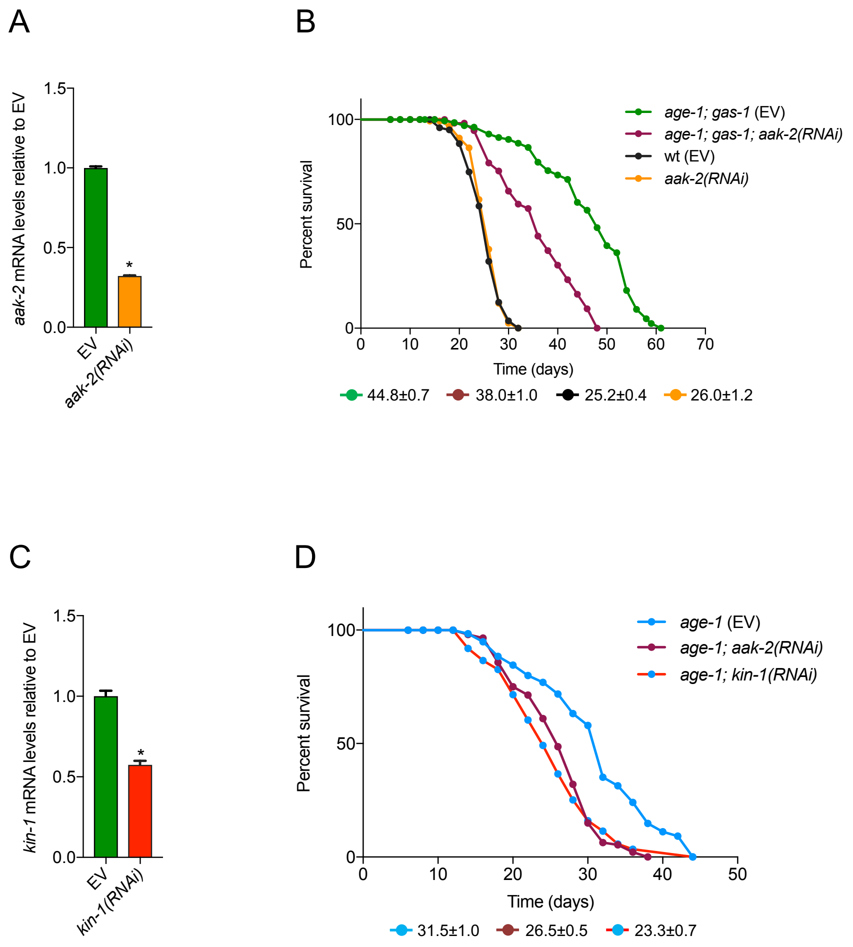

Appendix Figure S3

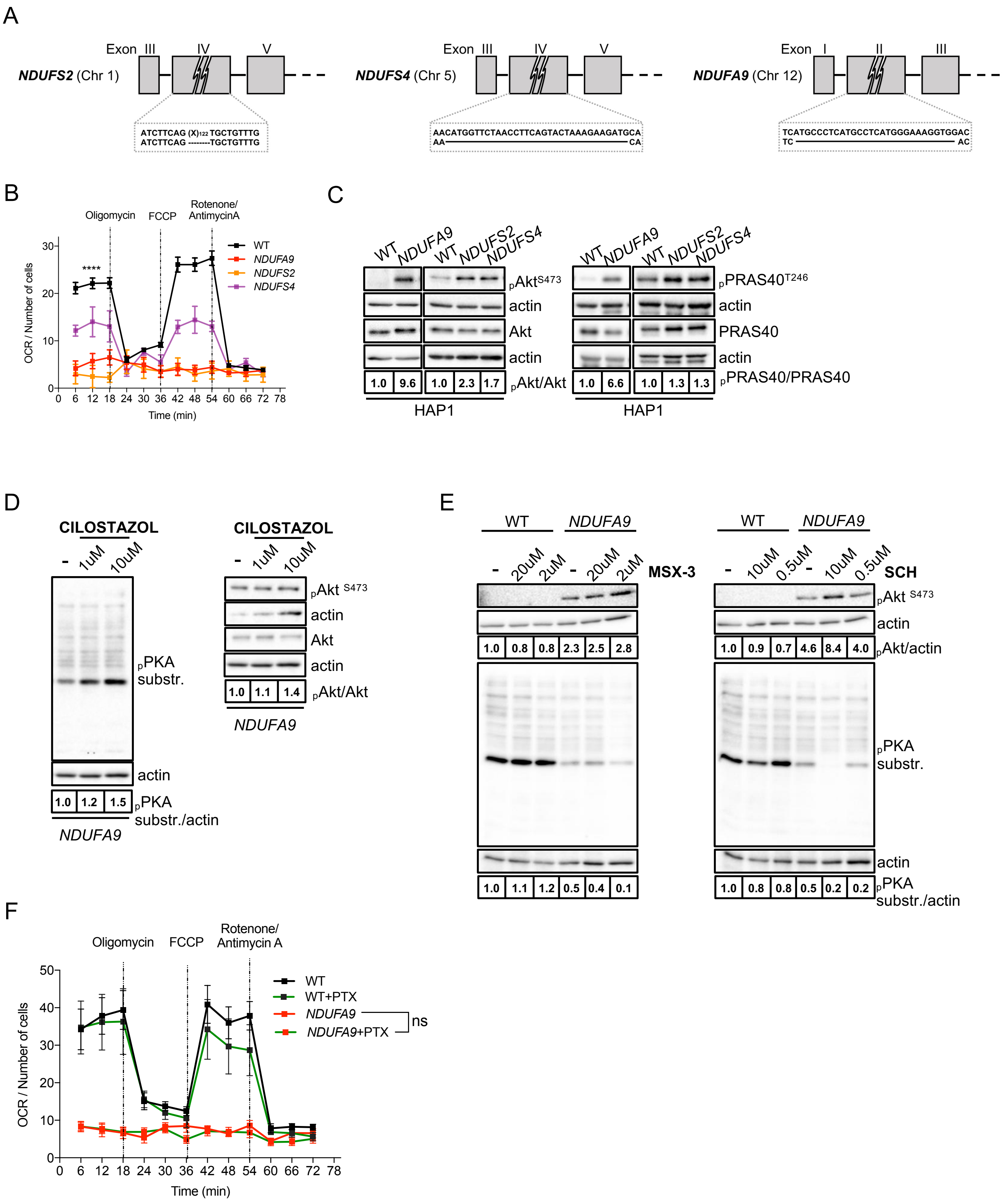

Appendix Figure S4

APPENDIX FIGURE LEGENDS

Appendix Figure S1. IIS inhibition is beneficial to nematodes carrying mitochondrial lesions (related to Figure 1).

(A) Overrepresented canonical pathways in phosphoproteomic analysis in *gas-1* compared to wt as calculated by IPA (FDR<20%, |z-score| >1).

(B) Volcano plot of differentially phosphorylated proteins in *gas-1(fc21)* mutants compared to wt. Cutoffs: 20% FDR, $|\log_2(\text{fold change})|>0.345$. In red: proteins significantly differentially phosphorylated. In blue: significantly differentially phosphorylated proteins that reverted to wt levels in *age-1; gas-1* compared to *gas-1* mutants.

(C) Representative lifespan curve of *pdk-1, gas-1* and *pdk-1; gas-1* mutants.

(D) Representative lifespan analysis of *gas-1* and *daf-16; gas-1*.

(E) qRT-PCR of *wah-1* in *age-1* mutants grown on bacteria carrying an empty vector (EV) or expressing *wah-1* RNAi (normalized to EV, n=3). Error bars: mean ± SEM, *****p* value< 0.0001, unpaired *t* test.

(F) Representative lifespan analysis of *age-1* mutants fed with empty vector (EV) or *wah-1* RNAi.

(G) Confocal images of representative mitochondrial morphology in muscle cells of L4 *age-1; gas-1* mutant worms grown on EV bacteria or expressing *fzo-1*. Scale bar: 5 µm.

(H) qRT-PCR of *fzo-1* in worms fed with empty vector (EV) or *fzo-1* RNAi to indicate downregulation efficiency. Error bars: mean ± SEM, n=3, **p* value< 0.05, Mann Whitney U test.

(I) Representative lifespan of *age-1* mutants fed with empty vector (EV) and *fzo-1* RNAi.

Data information: For all lifespan curves (C, D, F, I) average median lifespans ± SEM from all replicates (*gas-1* n=8; *pdk-1* n=2; *pdk-1; gas-1* n=2; *daf-16; gas-1* n=2; *age-1* (EV) n=4; *age-1* (*wah-1* RNAi) n=3; *age-1* (*fzo-1* RNAi) n=2) indicated beneath representative curves.

Appendix Figure S2. *age-1; gas-1* mutant animals have dynamic carbohydrate metabolism (related to Figure 2).

A) STRING analysis of differentially expressed proteins from TMT/iTRAQ scan (20% FDR, |$\log_2$(fold change)| > 0.47 Thickness of lines correlated to level of confidence of interaction.

(B) IPA prediction of glucose metabolism in in *age-1; gas-1* versus *gas-1* mutants. In orange, pathways that are predicted to be upregulated, while double colors indicate processes where different pathways give different predictions within the same dataset.

(C, D) qRT-PCR of (C) *aagr-1* and *aagr-2* (normalized to wt) and of (D) *pck-1* and *pck-2* (normalized to wt). Error bars: mean ± SEM, n=6. ****p value< 0.0001, ***p value< 0.001, **p value< 0.01, *p value< 0.05, ns= non-significant, Kruskal-Wallis with Dunn's post hoc test. wt= wild type, *a*= *age-1*, *g*= *gas-1*, *a; g*= *age-1; gas-1*.

Appendix Figure S3. AMPK and PKA are required for the lifespan extension of *age-1; gas-1* mutant animals (related to Figure 3).

(A) qRT-PCR of *aak-2* in *age-1; gas-1* mutants grown on bacteria carrying an empty vector (EV) or expressing *aak-2* RNAi (normalized to EV). Error bars: mean ± SEM, n=3, *p value< 0.05, Mann Whitney U test.

(B) Representative lifespan analysis of wt and *age-1; gas-1* mutant worms fed with empty vector (EV) or *aak-2* RNAi.

(C) qRT-PCR of *kin-1* in *age-1; gas-1* nematodes fed with empty vector (EV) or *kin-1* RNAi. Error bars: mean ± SEM, n=3, *p value< 0.05, Mann Whitney U test.

(D) Representative lifespan analysis of *age-1* mutants fed with empty vector (EV), *aak-2* and *kin-1* RNAi.

Data information: For all lifespans (B and D) average median lifespans ± SEM from all replicates (wt (EV) n=4; wt (*aak-2* RNAi) n=3; *age-1; gas-1* (EV) n=6; *age-1; gas-1* (*aak-2* RNAi) n=3) indicated beneath representative curves.

Appendix Figure S4. Complex I deficient HAP1 cells display hyperactive IIS (related to Figure 4).

(A) Schematic representation of the genetic deletions within the *NDUFS2*, *NDUFS4* and *NDUFA9* genes. The indicated loci were deleted using the CRISPR/Cas9 method. DNA sequencing confirmed the deletions in the corresponding loci.

(B) OCR in HAP1 cells lacking complex I subunit NDUFA9, NDUFS4 or NDUFS2 (normalized to no. cells/well). Error bars: mean ± SEM; n=9. Curves show one

representative experiment. ****p value< 0.0001(basal respiration compared to WT); one-way ANOVA, Dunnett's post-hoc correction.

(C) Immunoblot analyses of phospho-Akt (S473), pan Akt, phospho-PRAS40 (T246) and total PRAS40 in WT and complex I deficient HAP1 cells. Numbers represent band densitometries across 2-3 biological replicates.

(D) Western blot analysis of PKA phospho-substrates in *NDUFA9* KO HAP1 cells after treatment with the PDE inhibitor cilostazol for 4 h (actin loading control). For (D) and (E), numbers represent band densitometries of the representative immunoblots.

(E) Immunoblot analysis of WT and *NDUFA9* KO HAP1 cells after treatment with the adenosine receptor antagonists MSX-3 and SCH58261 (SCH) for 4 h (actin loading control).

(F) Oxygen consumption rate (OCR) in WT and *NDUFA9* KO HAP1 cells, following 24 h treatment with vehicle (DMSO) or PTX (normalized to no. cells/well). One representative curve shown, mean ± SEM, n=5, ns= non-significant, Mann Whitney U test.
