# Supplementary figures and images for: Multi‐omics identify xanthine as a pro‐survival metabolite for nematodes with mitochondrial dysfunction
Source: EMBO J. 2019 Feb 22;38(6):e99558. doi: 10.15252/embj.201899558 (PMC6418696; doi:10.15252/embj.201899558)

Appendix Figure S4C

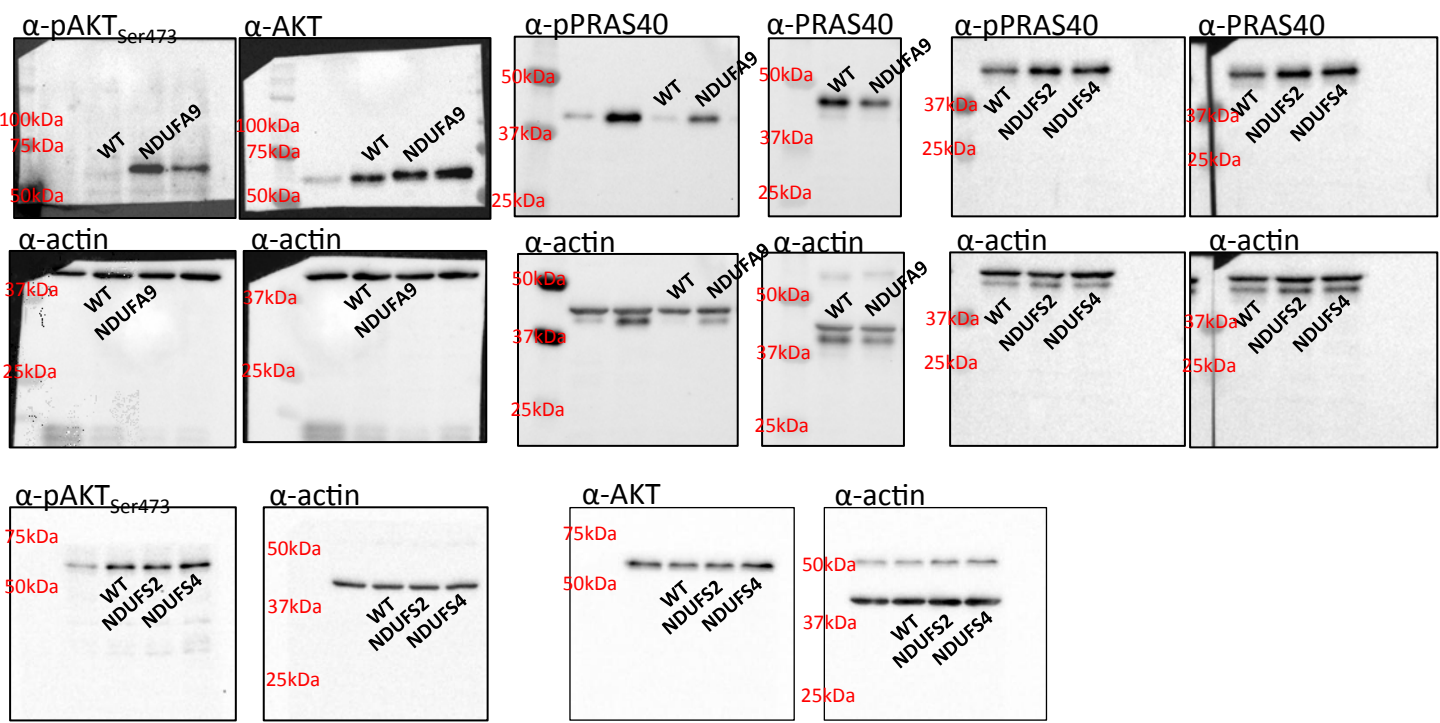

Appendix Figure S4D

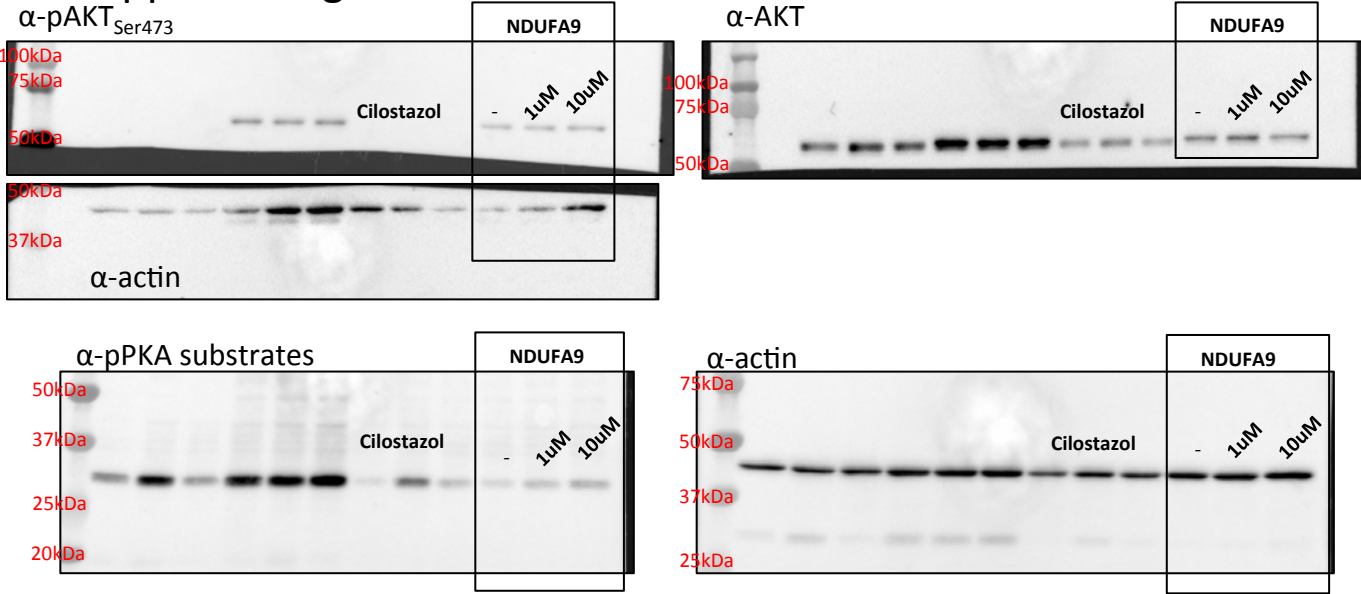

Appendix Figure S4E

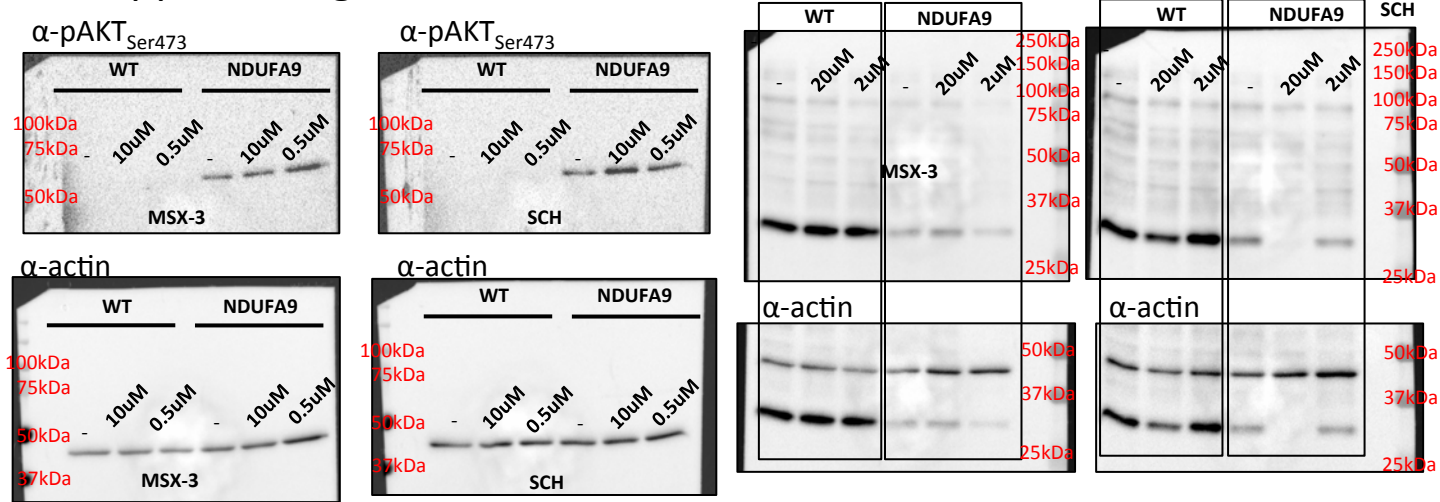

Supplement: Supplementary file 4 — Source Data for Appendix [file EMBJ-38-e99558-s007.pdf]

# Figure 3C

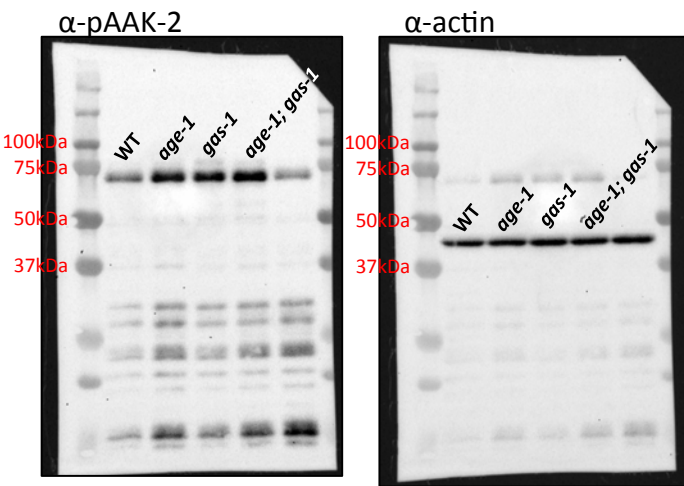

# Figure 3E

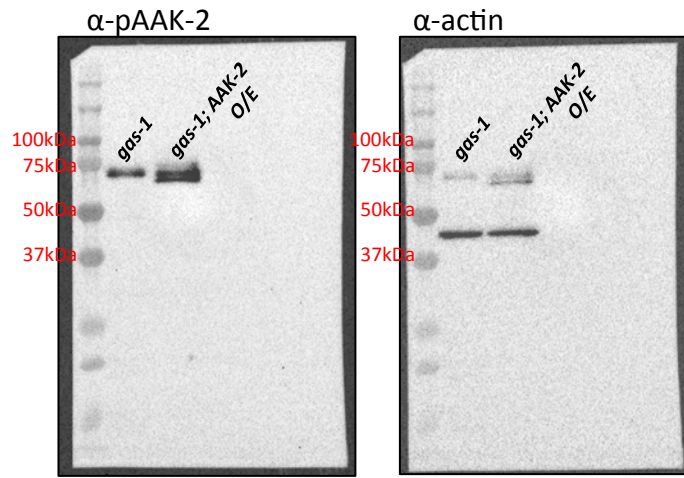

# Figure 3F

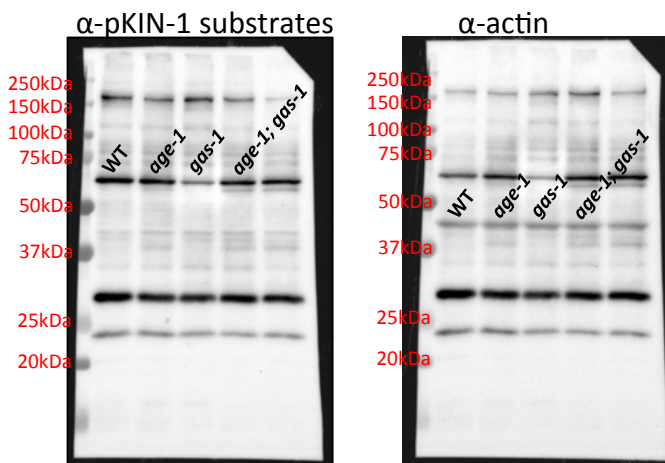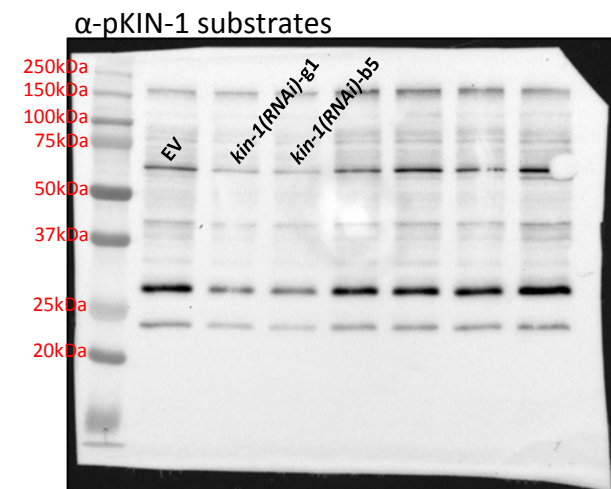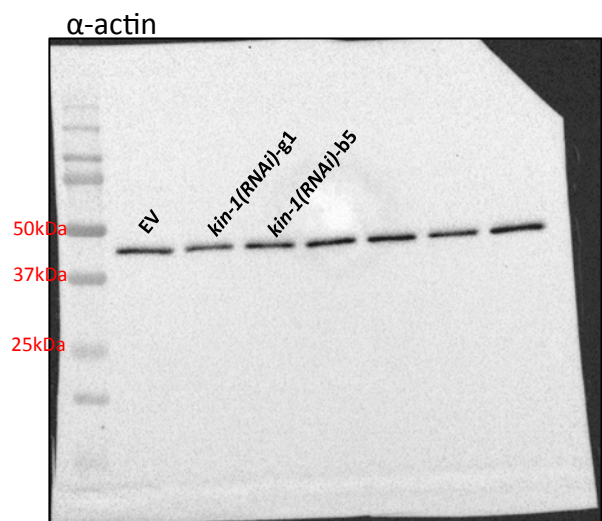

Supplement: Supplementary file 6 — Source Data for Figure 3 [file EMBJ-38-e99558-s004.pdf]

$\alpha$ -pAKT<sub>Ser473</sub>

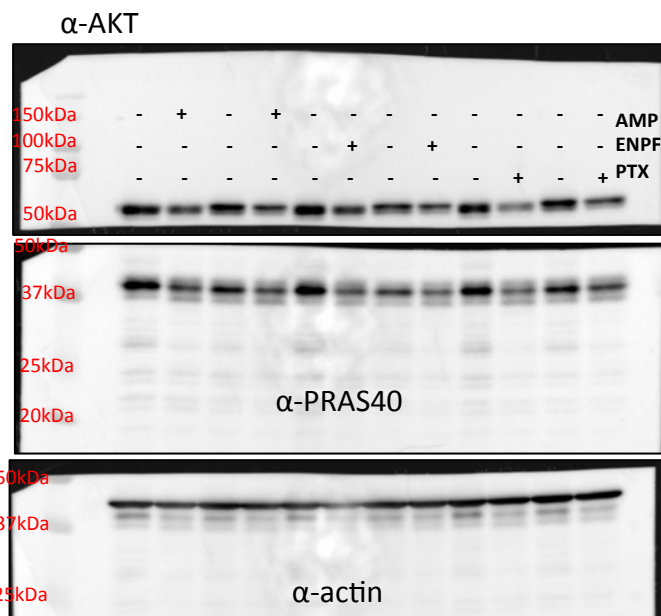

$\alpha$ -pPKA substrates

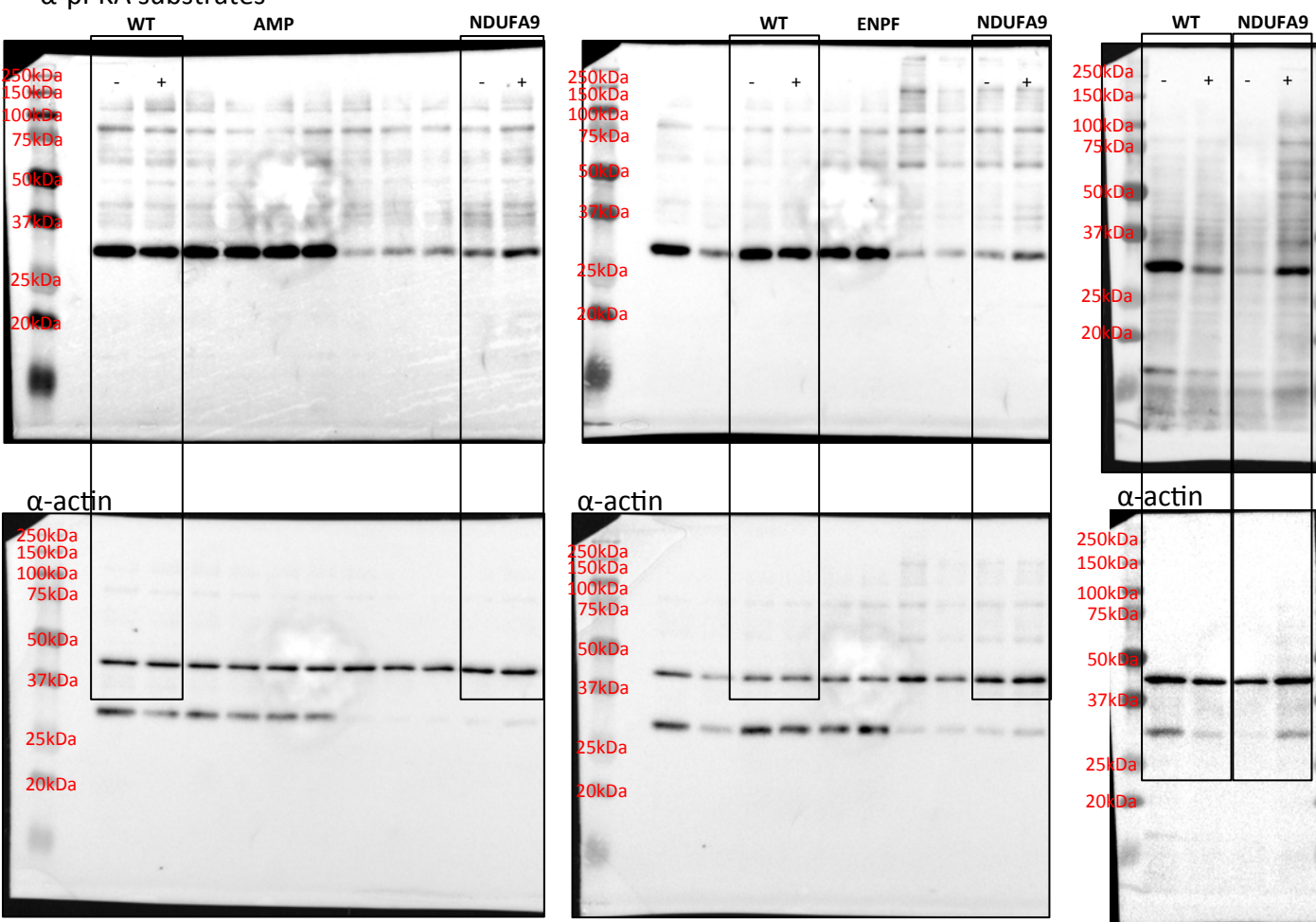

Supplement: Supplementary file 7 — Source Data for Figure 4 [file EMBJ-38-e99558-s005.pdf]

Figure 5E

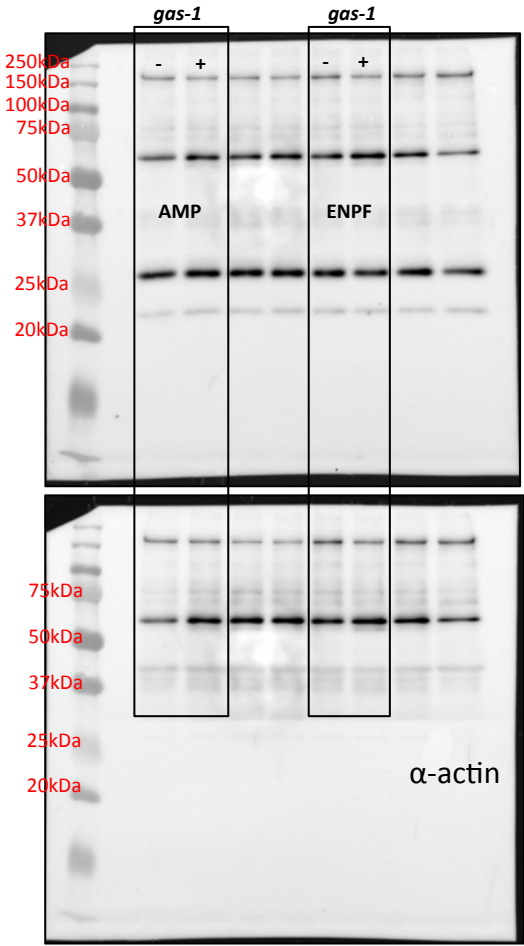

$\alpha$ -pKIN-1 substrates

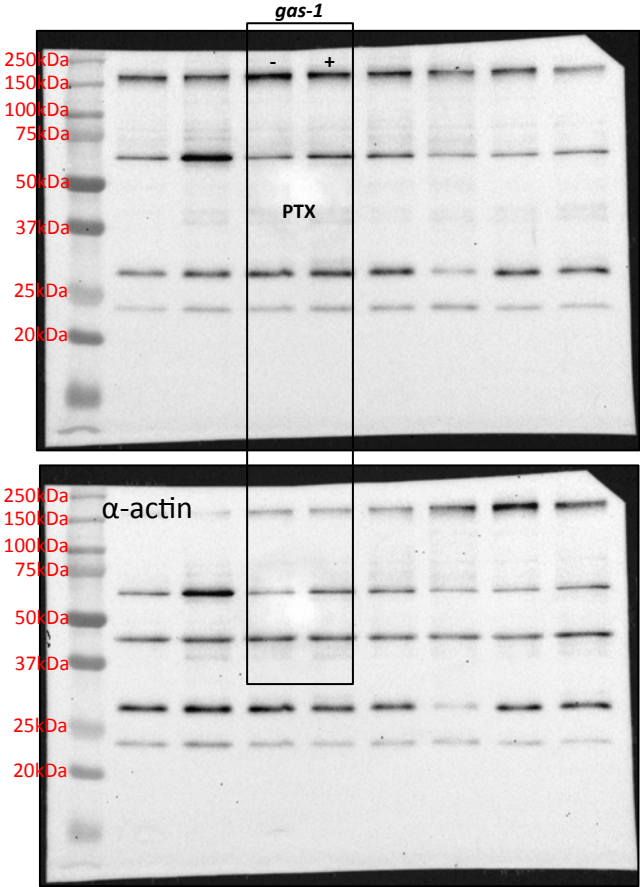

Figure 5F

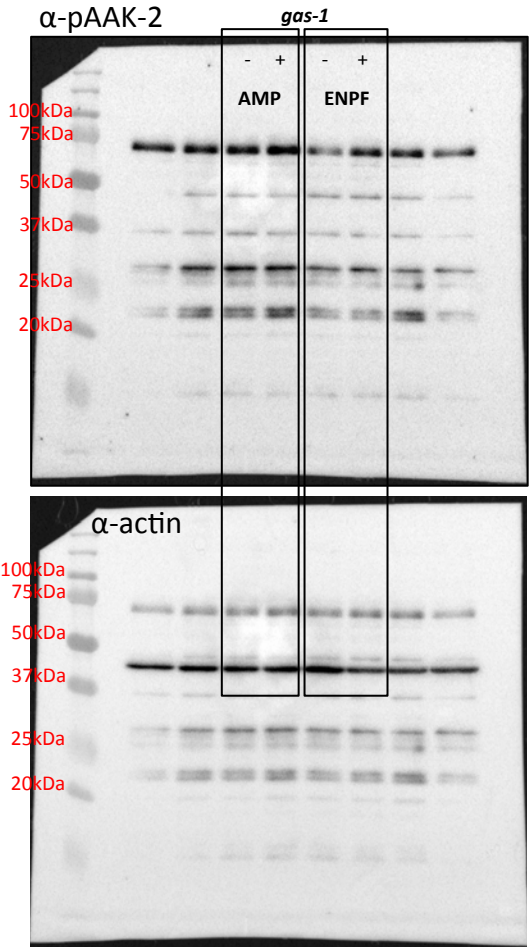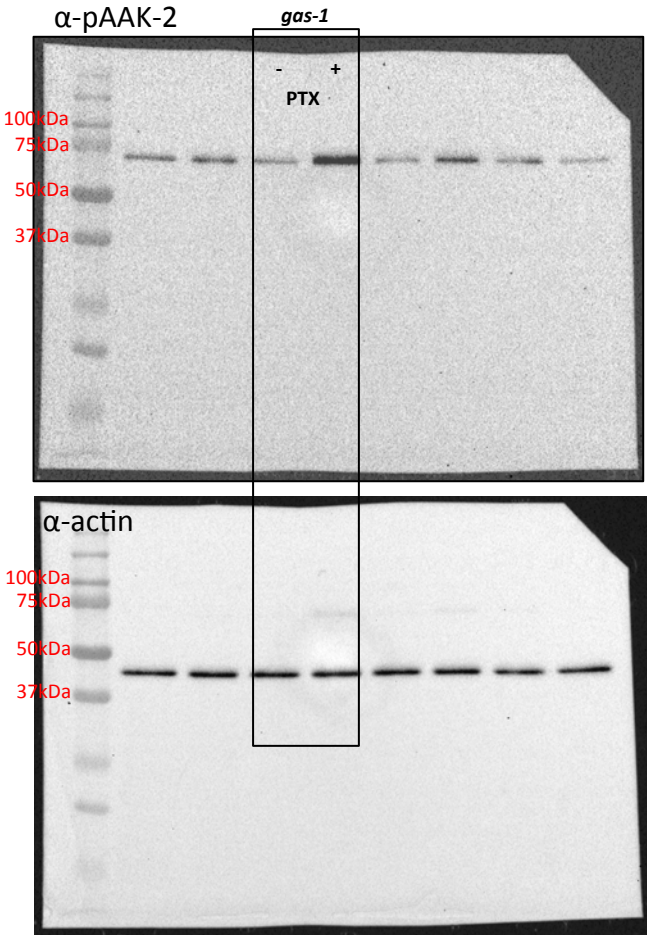

Supplement: Supplementary file 8 — Source Data for Figure 5 [file EMBJ-38-e99558-s006.pdf]
